# Helminth exposure influences Th17 plasticity, suppressing inflammatory and promoting regulatory activity by Th17 lineage cells

**DOI:** 10.3389/fimmu.2026.1767639

**Published:** 2026-04-28

**Authors:** Ahmed Metwali, Sarah Winckler, Xiaoqun Guan, M. Nedim Ince, David E. Elliott

**Affiliations:** 1 Internal Medicine, Iowa City Veterans Administration Health Center, Iowa City, IA, United States; 2 Department of Internal Medicine, Carver College of Medicine, University of Iowa, Iowa City, IA, United States

**Keywords:** colitis, Foxp3, helminth, IFN-γ, IL-10, Th17, Treg

## Abstract

**Introduction:**

Many autoimmune and inflammatory-mediated diseases are driven by pathogenic Th17 responses. Infection with parasitic worms (helminths) alters host immune responses, suppresses Th17 activity and can inhibit pathogenic inflammation. Instead of being terminally differentiated, Th17 cells are plastic and can assume highly pathogenic Th1-like function or more regulatory Tr1/Treg-like function. We investigated if helminth infection influences this Th17 plasticity.

**Methods:**

Lymphocytes from Th17-reporter mice permanently express eYFP if they previously transcribed IL-17. Using these mice, we examined if exposure to the intestinal helminth *Heligmosomoides polygyrus* bakeri altered the *in vitro* and *in vivo* regulatory activity of Th17 lineage cells.

**Results:**

We found that exposure intestinal helminths alter the Th17 compartment inhibiting development of Th1-like Th17 cells and promoting development of Tr1/Treg-like cells from the Th17 lineage. Furthermore, Th17 lineage cells from helminth-infected mice suppressed T cell proliferation and inhibited T cell-transfer colitis.

**Discussion:**

Thus, intestinal helminth-exposure not only reduces the frequency of Th17 cells in the MLN cell population, but in addition, changes the behavior of the remaining Th17-lineage cells to function more like classical T regulatory cells.

## Introduction

Industrialized countries that enjoy highly hygienic lifestyles are in the midst of an epidemic of autoimmune and immune-mediated inflammatory disease (1). Many environmental changes occur with socioeconomic advancement, but one with immunologic consequence is loss of routine exposure to helminthic parasites (2, 3). Helminths (parasitic worms) are complex multicellular organisms that alter and evade host immune responses. Infection with helminths increases immune regulatory cell function, suppresses pro-inflammatory cytokine release, and can reduce pathology in animal models of inflammation (4). This ability to modify host immunity supports the hypothesis that the lack of previously ubiquitous helminth exposure contributes to the epidemic of immune-mediated disease.

Many autoimmune and immune-mediated inflammatory diseases are driven by Th17 cell activity (5). While appropriate Th17 cell function is important for intestinal homeostasis (6), excessive or pathogenic Th17 activity results in inflammatory bowel disease (6–8). Indeed, the behavior of Th17 cells is not uniform and rigid but is heterogeneous and plastic. Although once thought to be terminally differentiated, Th17 cells are now known to be plastic and able to further develop into highly inflammatory Th17/Th1-like cells or more regulatory Th17/Treg-like cells (9). We and others have shown that helminth infection suppresses Th17 cell activity and reduces pathologic inflammation (10, 11). Using Th17-lineage reporter mice, we now show that exposure to the murine helminth *Heligmosomoides polygyrus bakeri* also skews Th17 plasticity away from an inflammatory and to a regulatory profile.

## Materials and methods

### Mice

Rag1^−/−^ mice on a C57BL/6 background, C57BL/6 CD45.2+ wild type, C57BL/6 CD45.1+ wild type, IL-17a^Cre^, and R26R-eYFP were purchased from The Jackson Laboratory, Bar Harbor, ME. IL-17a^Cre^ mice were bred with R26R-eYFP mice to make heterozygous IL-17a lineage reporter mice. Genotyping was performed as per recommended protocols. Tissues were isolated from adult mice euthanized by CO_2_ narcosis followed by cervical dislocation as per recommended protocols. Breeding colonies of the wild type and mutant animals were maintained in specific pathogen-free facilities at the University of Iowa.

### Single-cell preparations used for transfer to Rag1^-/-^ mice and for *in-vitro* cultures

Single-cell suspensions of splenocytes or mesenteric lymph nodes (MLN) were prepared by exposing the tissue fragments to cycles of suction and expulsion through a 1-ml syringe in RPMI 1640 medium (Life Technologies, Grand Island, NY). Red blood cells were lysed by exposing the cells briefly to sterile hypotonic shock in a 50-ml tube and washing twice in RPMI 1640. The cells were passed through a 100-μm nylon cell strainer (BD Falcon 352360, MA, USA) to exclude the remaining tissue fragments. eYFP-expressing MLN cells from helminth-naïve or 2-week-infected Th17 lineage reporter mice were isolated by flow cytometry/sorting. Briefly, we sorted eYFP-positive cells from ~1.5 × 10^8^ viable dispersed MLN cells using a Becton and Dickson Cytek Aurora Cell Sorter with 355, 405, 488, 561, and 640 nm lasers at the Flow Cytometry Core Facility at the University of Iowa, Iowa City IA. Viable cells were identified using eFluor 660 (e-Bioscience, San Diego, CA). The average yield of eYFP-positive sorted cells was 1 × 10^5^ to 1 × 10^6^ cells with 95% viability.

For T-cell transfer experiments, negatively selected T cells were isolated from dispersed spleen cells using a cocktail of antibodies to label B cells, monocytes, CD25^+^ cells, and granulocytes. These antibodies were prepared in our laboratory from clones B220, Mac1, PC61.5, and GR-1, obtained from the American Type Culture Collection (ATCC), USA, by using ammonium sulfate precipitation and dialysis of hybridoma supernatants. We used microbeads coated with sheep anti-rat IgG to remove antibody-labeled cells using a magnet from Invitrogen Dynal AS, Oslo, Norway, according to the manufacturer’s specifications. Purified CD25neg T cells (5 × 10^6^ cells in 1 ml of PBS) were injected intraperitoneally into each Rag1^−/−^ recipient mouse.

Lamina propria mononuclear cells (LPMCs) were isolated from colons that were extensively washed with RPMI. The intestines were opened longitudinally, cut into 5-mm pieces and then incubated in 0.5 mM EDTA in calcium- and magnesium-free Hanks’ for 20 min at 37 °C with shaking to release intraepithelial lymphocytes and epithelial cells. Tissues were then incubated for 20 min at 37 °C in 20 ml RPMI containing 1 mg/ml collagenase (Sigma-Aldrich, St. Louis, MO United States of America), 10% FCS, 25 mM HEPES buffer, 2 mM L-glutamine, 5 × 10^−5^ M β-mercaptoethanol, 1 mM sodium pyruvate, 100 U/ml penicillin, 5 mg/ml gentamycin, and 100 mg/ml streptomycin. After digestion, cells were freed by gentle mechanical disruption using a 1-ml syringe and quickly sieved through a wet, loosely packed nylon wool column. After washing, cells (up to 2 × 10^7^) were layered onto a Percoll 30:70% gradient and then centrifuged at 2200 × G at room temperature for 20 min. The LPMC were collected from the 30:70 interface. Cell viability (>80%) was determined by Trypan blue exclusion.

### Rag1^-/-^ model of transfer colitis

Rag1^-/-^ mice of C57BL/6 background were reconstituted with negatively selected splenic CD25neg T cells (5 × 10^6^ cells/mouse) from the same gender. For some experiments, negatively selected splenic CD25neg T cells from CD45.1 positive mice were admixed with 3 × 10^4^ eFYP+ MLN T cells prior to injection (at 5.3 × 10^6^ cells/mouse) into Rag1^-/-^ recipients. To induce and synchronize colitis, three independent groups of two to five mice each of reconstituted Rag1^−/−^ mice were treated with the NSAID, Piroxicam (P-5654, SIGMA), mixed in their ground feed NIH-31 diet 7013 from Harlan, WI, USA, for 2 weeks. 40 mg/250 g of ground feed was used during the first week, and 60 mg/250 g was used during the second week. To grade intestinal inflammation, colons were removed and opened, cleaned from debris, and the tissue placed on glass rods to produce a “Swiss roll,” fixed in 4% paraformaldehyde in PBS, then sectioned and stained with hematoxylin and eosin (H&E). The inflammation was scored, in a blinded fashion, from 0 to 4 using the following criteria: *grade 0*, no change from normal tissue; *grade 1*, patchy mononuclear cell infiltrates in the lamina propria; *grade 2*, more uniform mononuclear cell inflammation involving both the epithelium and lamina propria; this was accompanied by minimal epithelial hyperplasia and slight-to-no depletion of mucous from goblet cells; *grade 3*, some epithelial and muscle hypertrophy with patchy lymphocytic infiltrates extending into the muscle layers; there was mucus depletion and occasional crypt abscesses and epithelial erosions; *grade 4*, transmural inflammation involved most of the intestinal section; lesions composed mostly of lymphocytes and some neutrophils, prominent thickening of both the epithelial and muscle layers, and mucus depletion with more frequent crypt abscesses (10). Colitic mice used in our experiments developed grade 3 and above colitis following piroxicam exposure.

### Mice inoculated with the parasitic helminth *Heligmosomoides polygyrus bakeri*

Mice were housed and handled according to National Institutes of Health guidelines and as approved by our Institutional Animal Care and Use Committee. Mice were inoculated with 150 third-stage infective ensheathed larvae (L3) of *H. polygyrus bakeri* (U.S. National Helminthological Collection no. 81930) by oral gavage.

### Cell cultures

For cytokine analysis and proliferation studies, cells were adjusted to 5 × 10^6^ cells/ml in RPMI 1640 containing 10% FCS, 25 mM HEPES buffer, 2 mM L-glutamine, 2β-ME, 1 mM sodium pyruvate, 100 U/ml penicillin, 5 mg/ml gentamicin, and 100 mg/ml streptomycin. Cell suspensions were dispensed in 200 μl/well into 96-well microtiter plates (Costar 3595 from Corning Incorporated, NY, USA). Cell cultures were stimulated with soluble anti-CD3 (1 μg/ml) prepared in our laboratory from clone 2C11 (ATCC CRL-1975), LPS from E. coli 055:B5 (10 μg/ml) L2637 from SIGMA or CpG, ODN1826 (0.6 μg/ml) from IDT, Coralville, Iowa, and were incubated at 37°C for 48h in 5% CO_2_ in air at 100% humidity in a closed incubator.

### Cytokine ELISA’s

Culture supernatants were harvested after spinning down the cells and transferred to new plates. The concentration of IL-17a, IFN-γ, IL-10, and IL-12p40 were determined by sandwich ELISAs. Briefly, plates were coated with monoclonal antibodies extracted from the following hybridomas. XMG 1.2 (αIFN-γ, DNAX Research Institute of Molecular and Cellular Biology, Palo Alto, CA), 2A5 (αIL-10, ATCC, CRL-2444), TC11-18H10.1 (αIL-17a, BioLegend, San Diego, CA United States of America), and C16.5 (αIL-12p40, hybridoma: a gift from Dr. G. Trinchieri, (Wistar Institute of Anatomy and Biology, Philadelphia, PA). For detection, R4.6A2-biotin (αIFN-γ, from e-Bioscience, USA), JESS-16E3-biotin (αIL-10, BioLegend, San Diego, CA United States of America), TC11-8H4-biotin (αIL-17a, BioLegend), and C17.8-biotin (αIL-12p40, BioLegend) were used. ELISA protocols were adapted from the recommended manufacturer’s directions. ELISA sensitivities were determined by using mouse recombinant cytokines as 30 pg/ml for IFN-γ, 30 pg/ml for IL-17a, 60 pg/ml for IL-10, and 30 pg/ml for IL-12p40.

### Flow cytometry

Lymph node cells were washed in RPMI, adjusted to 10^7^ cells/ml in FACS buffer (HBSS containing 1% FCS, 20 mM HEPES), and dispensed 100 μl/staining tube. Each tube received 1 μg of anti-Fcγ-R monoclonal antibody (Clone 2.4G2, ATCC) to block nonspecific binding, and then saturating amounts of conjugated m-Ab were added for 30 min at 4 °C. Monoclonal antibodies used in flow cytometry to stain the surface receptors, including CD4-FITC or APC (Clone GK1.5) and CTLA4-PE (Clone UC10-4B9), with the appropriate fluorochrome combinations, were purchased from BD-Pharmingen or e-Bioscience. Intra-cytoplasmic staining of IL-17a (e-Bioscience, San Diego, CA), IL-12/23p40 (BioLegend, San Diego, CA), IFN-γ, and Foxp3 using IL-17a-PE, IL-12/23(P40) Per-CP, IFN γ-APC (BD-Bioscience, San Diego, CA), and Foxp3-APC or PE (Clone FJK-16s, e-Bioscience #, San Diego, CA) in CD4^+^ T cells were performed according to the manufacturer’s instructions. MLN cells were stimulated overnight with phorbol myristate acetate and ionomycin. Cells were stained for surface receptors first, then fixed and stained for intracytoplasmic cytokines according to the manufacturer’s instructions. Staining of surface receptors and cytokines was done in parallel with irrelevant isotype control antibodies to ensure specificity and with fluorescence-minus-one analysis to determine gating (**Supplementary Figure 1**).

Following staining, cells were washed twice and resuspended in lymphocyte growth medium for analysis. Cell acquisition was done by a FACS Calibur flow cytometer, and analysis was done using FlowJo software (BD Biosciences, Mountain View, CA) in the flow cytometry core facility at the University of Iowa.

### Suppression of proliferation assays

Normal splenic lymphocytes were isolated from C57BL/6 CD45.1+ mice and labeled with carboxyfluorescein diacetate succinimidyl ester (CFSE, eBioscience) (12). CFSE-labeled splenic lymphocytes were added to eYFP^+^ sorted MLN cells at a ratio of 2:1 and then cultured for 48h in the presence of anti-CD3 mAb as described above. The following culture cells were stained for CD45.1 expression (A20, BioLegend) and then examined on a Becton Dickinson LSR II, and data were analyzed by using FlowJo software (13).

### Statistical analysis

Unless otherwise stated, all data are expressed as means ± SE of three experiments with five mice in each experimental group. Statistically significant differences in cytokine production between groups were determined by using the Student t-test (Microsoft Excel). Statistically significant differences in the suppression of cell proliferation (as measured by CSFE dilution) were determined with Spearman’s correlation test. Statistically significant differences in histology scores between groups were determined by using the Mann–Whitney U test [Real Statistics Resource Pack (Release 5.4)]. Values with differences of *p* < 0.05 were considered significant.

## Results

### Helminth infection reduces frequency of CD4+ Th17 lineage cells in murine mesenteric lymph nodes

**Figure 1:**
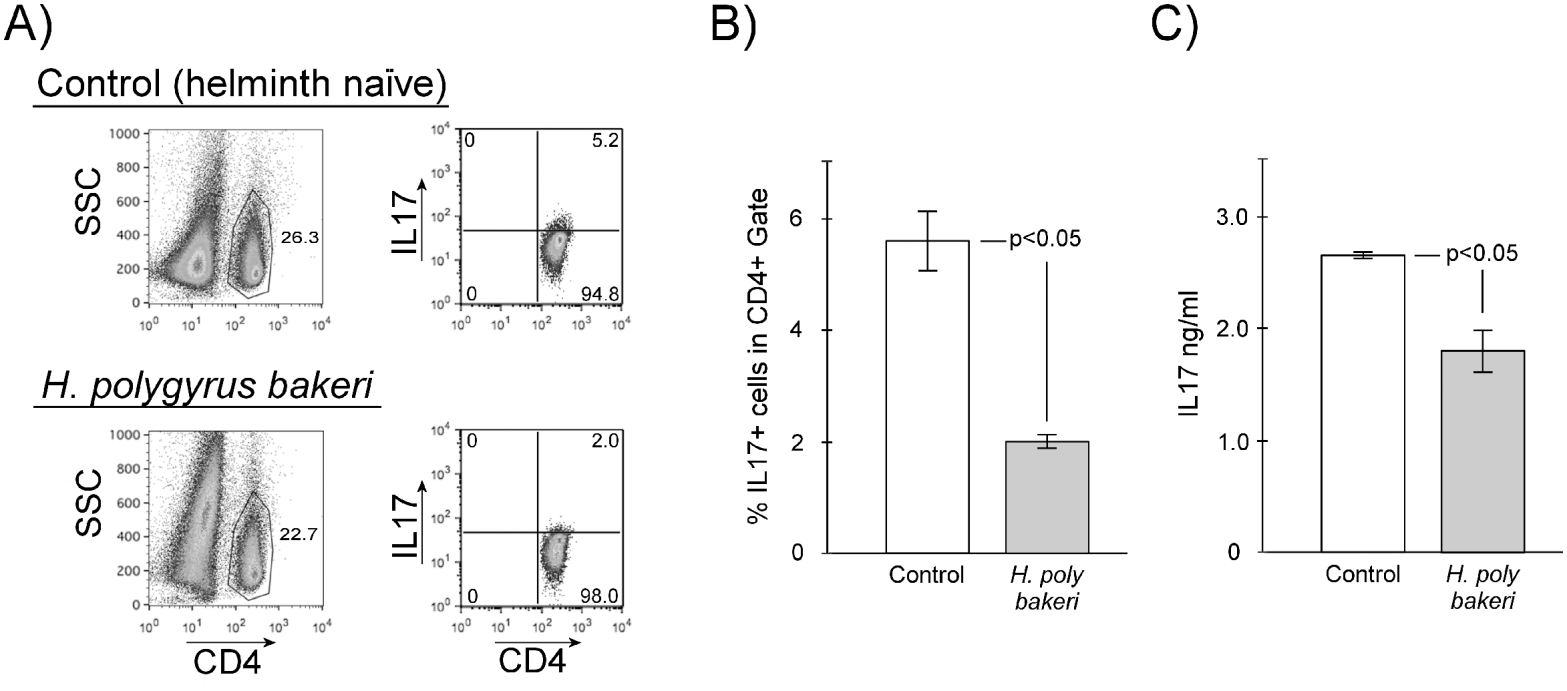
Effect of helminth exposure on IL-17a protein expression in CD4+ MLN cells from IL-17a lineage reporter mice. MLN cells were isolated from helminth naïve (Control) or *H. polygyrus bakeri*–infected mice then analyzed by flow cytometry for expression of intracellular IL-17a **(A, B)** or cultured in the presence of αCD3 for 48h then culture supernatants tested for IL-17a by ELISA **(C)**. **(A)** Representative flow cytometry showing gating for CD4+ and IL-17a expression by cells in that gate. **(B, C)** Data are means ± S.E. from three independent experiments.

**Figure 2:**
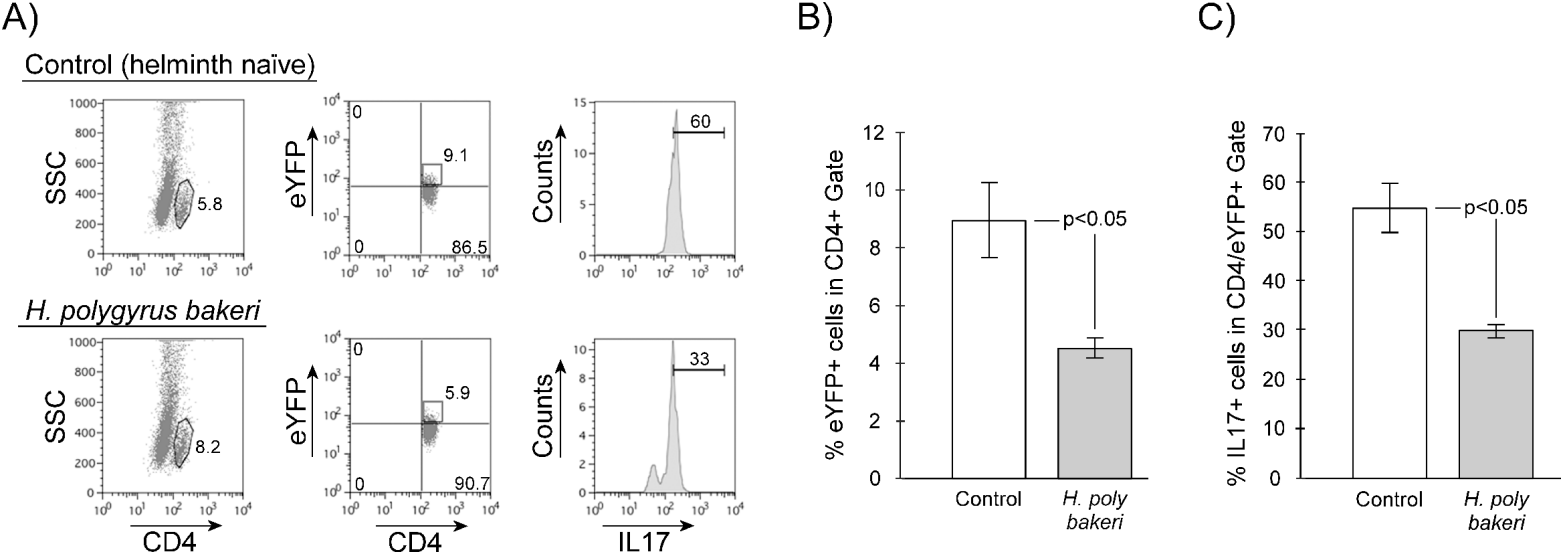
Helminth exposure effects frequency of CD4+ eYFP + cells and the level of IL-17a expression in MLN cells from IL-17a lineage reporter mice. MLN cells were isolated from helminth naïve (Control) or *H. polygyrus bakeri*-infected mice then analyzed by flow cytometry for expression of both eYFP and intracellular IL17a. **(A)** Representative flow cytometry showing gating for CD4+ eYFP+ cells and IL-17a expression by cells in that gate. **(B)** and **(C)** Data are means ± S.E. from three independent experiments.

Previously, we showed that enteric helminth infection with *Heligmosomoides polygyrus bakeri* (Hpb) significantly decreases the frequency of mesenteric lymph node (MLN) CD4+ Th17 cells (11) as measured by cellular expression of IL-17. We repeated these experiments using IL-17 lineage reporter mice. Th17 lineage reporter mice were generated by breeding IL-17a^cre^ C57BL/6J mice (JAX 035717) that express Cre driven by the native IL-17a promoter (14) with a reporter mouse R26R-eYFP C57BL/6J mice (JAX 006148) that will permanently express enhanced yellow fluorescent protein after a *loxP*-flanked stop sequence is excised by Cre (15). These mice are heterozygous for intact IL-17a gene expression. We infected Th17 lineage reporter mice with *Hpb* L3 larvae. Two weeks after initiation of infection, we euthanized the mice and evaluated their MLN CD4+ cells for IL-17a and eYFP expression. Like wild-type mice (11), infection with Hpb results in a significant decrease in the frequency of IL-17a-expressing MLN CD4+ cells (**Figure 1B**, helminth naïve = 5.60 ± 0.53%, *Hpb*-infected = 2.0 ± 0.12%, mean ± SE, *p* < 0.05) and in their mitogen-stimulated production of IL-17a (**Figure 1C**, helminth-naïve = 2.66 ± 0.03 ng/ml, *Hpb*-infected = 1.80 ± 0.10 ng/ml, mean ± SE, *p* < 0.05). Likewise, after helminth infection, there is also a decrease in the frequency of MLN cells that express eYFP (Th17 lineage cells, **Figure 2B**, helminth naïve = 8.95 ± 1.30%, *Hpb*-infected = 4.51 ± 0.34%, mean ± SE, *p* < 0.05). With or without *Hpb* infection, the percentage of Th17 lineage cells in the MLN compartment is greater than the percentage of cells actively expressing IL-17a protein as measured by intracellular flow cytometry (**Figure 2C**). Helminth infection significantly decreased the percentage of IL-17a-expressing cells within the already depressed Th17 lineage (**Figure 2C**, helminth naïve = 54.87 ± 4.98%, *Hpb*-infected = 29.09 ± 1.35%, mean ± SE, *p* < 0.05).

### Helminth infection skews IFNγ and IL-10 expression by CD4+ Th17 lineage cells in murine MLN

**Figure 3:**
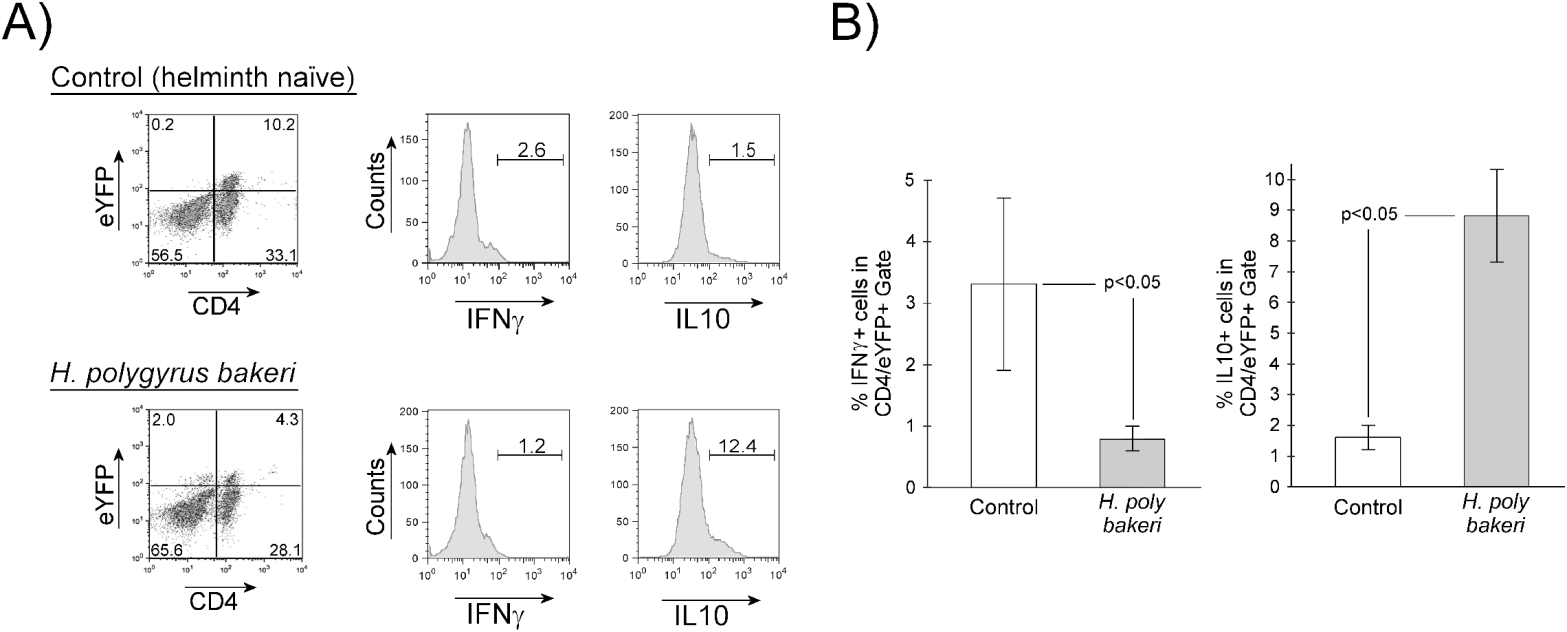
Helminth exposure alters IFN-γ and IL-10 expression by MLN CD4+ IL17a lineage T cells. MLN cells were isolated from helminth naïve (Control) or *H. polygyrus bakeri*–infected mice then analyzed by flow cytometry for expression of both eYFP and intracellular IFN-γ or IL-10. **(A)** Representative flow cytometry showing gating for CD4+ eYFP+ cells and IFN-γ or IL-10 expression by cells in that gate. **(B)** Data are means ± S.E. from three independent experiments.

Th17 cells display plasticity. Highly inflammatory Th17 cells express IFN-γ, and non-inflammatory or less inflammatory Th17 cells express IL-10 (9). Helminth infection significantly reduces the frequency of Th17 lineage cells in the MLN population, decreases the percentage of IL-17a-producing cells within the Th17 lineage, and may also alter their plasticity. We examined the MLN Th17 lineage cells to determine if helminth infection affects their IFN-γ or IL-10 expression. As shown in **Figure 3A**, Hpb infection decreases the frequency of MLN cells that express eYFP. Gating on the eYFP-expressing CD4+ cells shows that IFN-γ expression was suppressed (**Figure 3B** helminth naïve = 3.22 ± 1.40%, *Hpb*-infected = 0.79 ± 0.20%, mean ± SE, *p* < 0.05) and IL-10 expression augmented (**Figure 3B** helminth naïve = 1.61 ± 0.40%, *Hpb*-infected = 8.82 ± 1.51%, mean ± SE, *p* < 0.05) within MLN Th17 lineage cells.

### Helminth infection significantly increases expression of Foxp3 in the Th17 lineage but the expansion of MLN Foxp3+ T cells is not due solely to this increase

**Figure 4:**
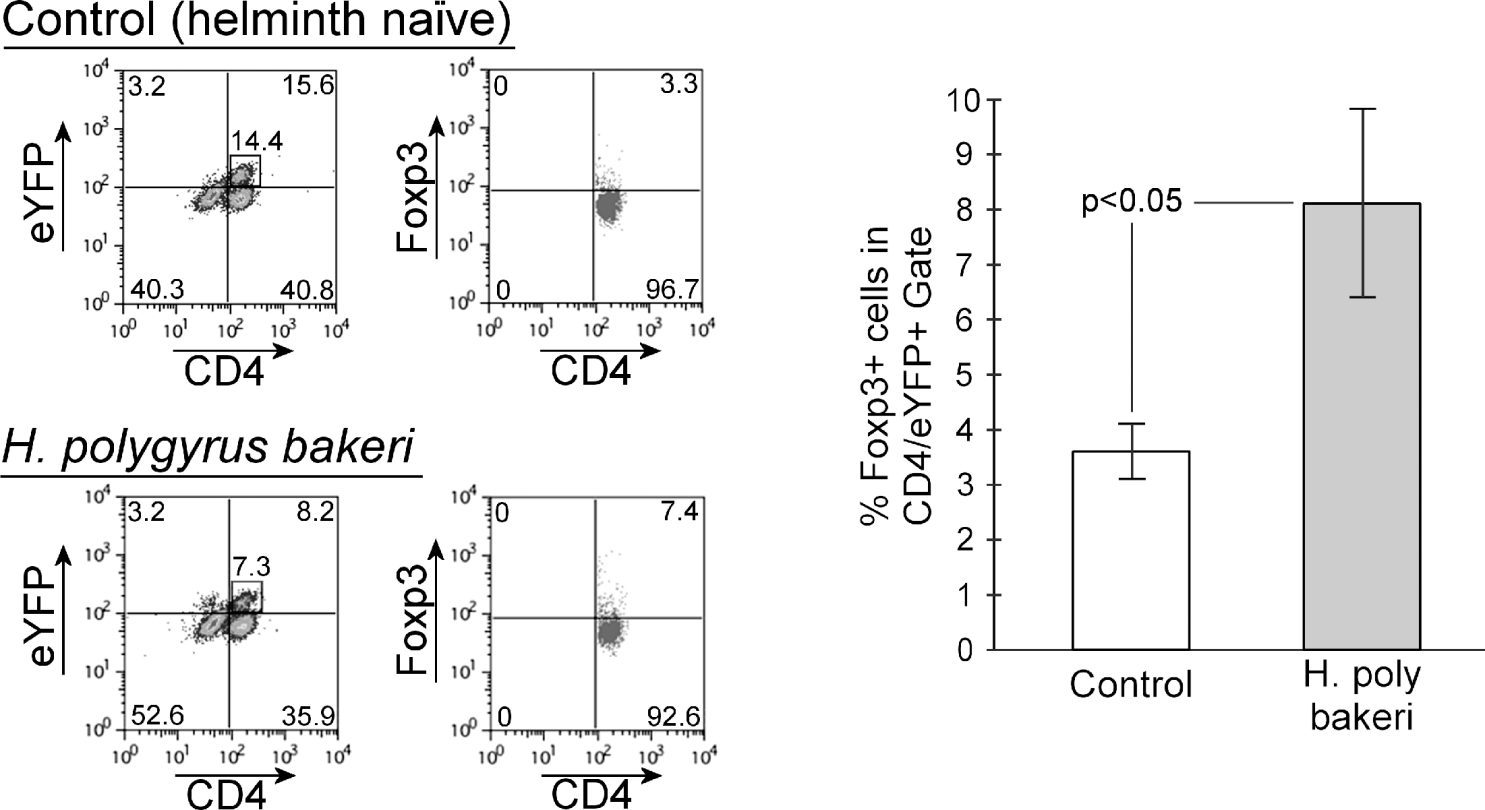
Helminth exposure alters Foxp3 expression in the Th17 cell lineage compartment. MLN cells were isolated from helminth naïve (Control) or *H. polygyrus bakeri*-infected mice then analyzed by flow cytometry for expression of both eYFP and Foxp3. Bar graphs show means ± S.E. from three independent experiments.

**Figure 5:**
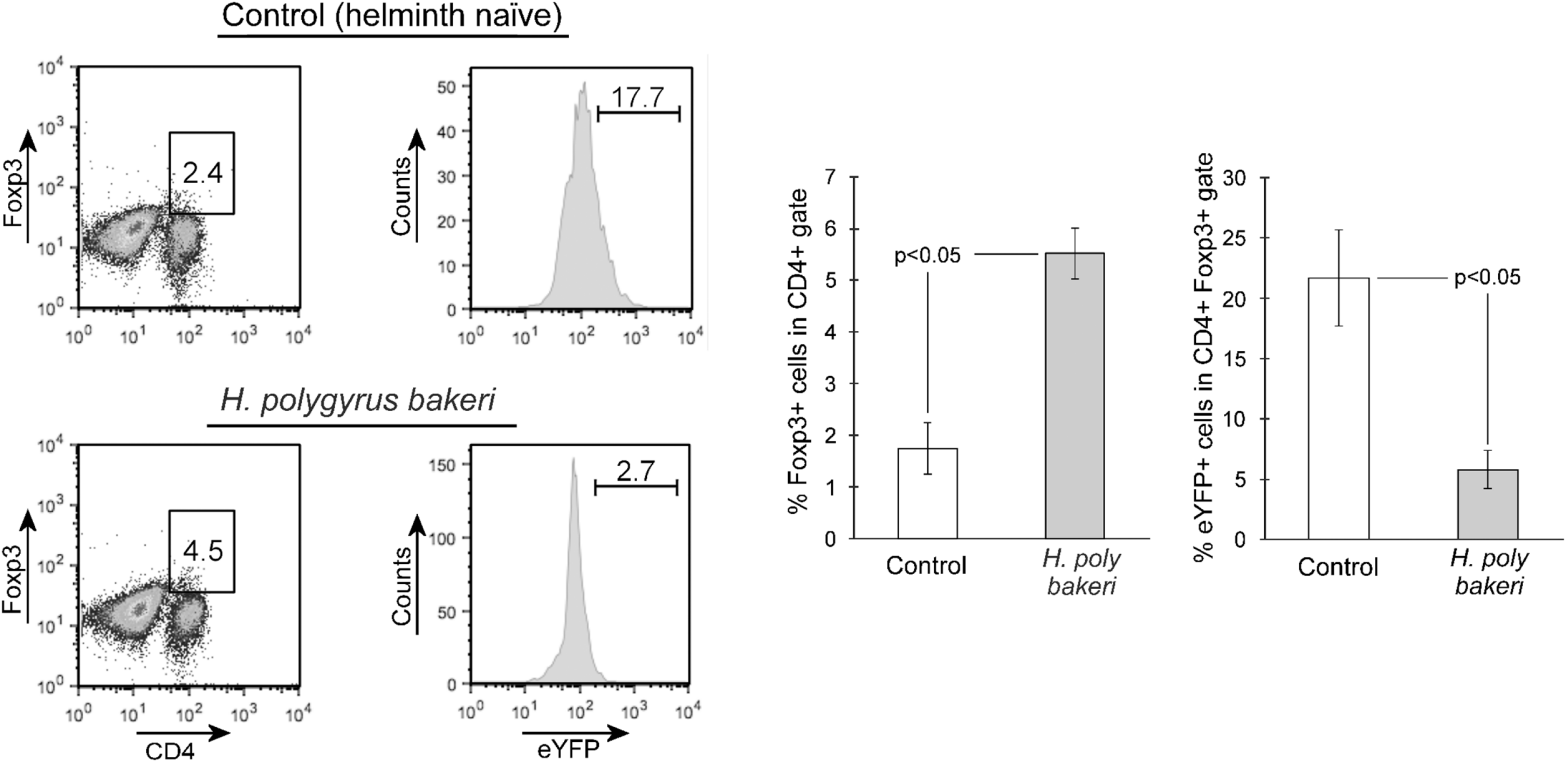
Helminth exposure reduces the frequency of Th17 lineage cells om the MLN CD4+Foxp3+ compartment. MLN cells were isolated from helminth naïve (Control) or *H. polygyrus bakeri*–infected mice then analyzed by flow cytometry for expression of CD4 and Foxp3. Cells in the CD4+Foxp3+ gate were then evaluated for expression of eYFP. Bar graph data are means ± S.E. from three independent experiments.

Infection with Hpb increases the frequency of CD4+ Foxp3+ MLN cells (16, 17). We examined if infection expands Foxp3 expression in Th17 lineage cells. Like the increase in IL-10 expression (**Figure 3**), helminth infection significantly augments the percentage of Th17 lineage MLN cells that express Foxp3 (**Figure 4**, helminth-naïve = 3.60 ± 0.50%, *Hpb*-infected = 8.11 ± 1.71%, mean ± SE, *p* < 0.05). We next tested to see if the expansion of Foxp3+ MLN cells following helminth infection was due, in part, to the augmentation of Foxp3+ cells in the Th17 lineage. As shown in **Figure 5**, infection significantly increases the percentage of MLN CD4+ Foxp3+ T cells (helminth naïve = 1.74 ± 0.50%, *Hpb*-infected = 5.54 ± 0.49%, mean ± SE, *p* < 0.05). In helminth-naïve mice, 21.67 ± 4.00% (mean ± SE) of the MLN CD4+ Foxp3+ cells also express eYFP, showing that at one time their IL-17a gene was actively expressed. After helminth infection, the percentage of Foxp3+ CD4+ MLN T cells that express eYFP decreases nearly fourfold to 5.66 ± 1.58% (mean ± SE, *p* < 0.05). Thus, the helminth infection-associated decrease in frequency of MLN Th17 lineage cells is also seen within the Foxp3+ compartment.

### Helminth infection augments suppressor cell activity in Th17 lineage cells

**Figure 6:**
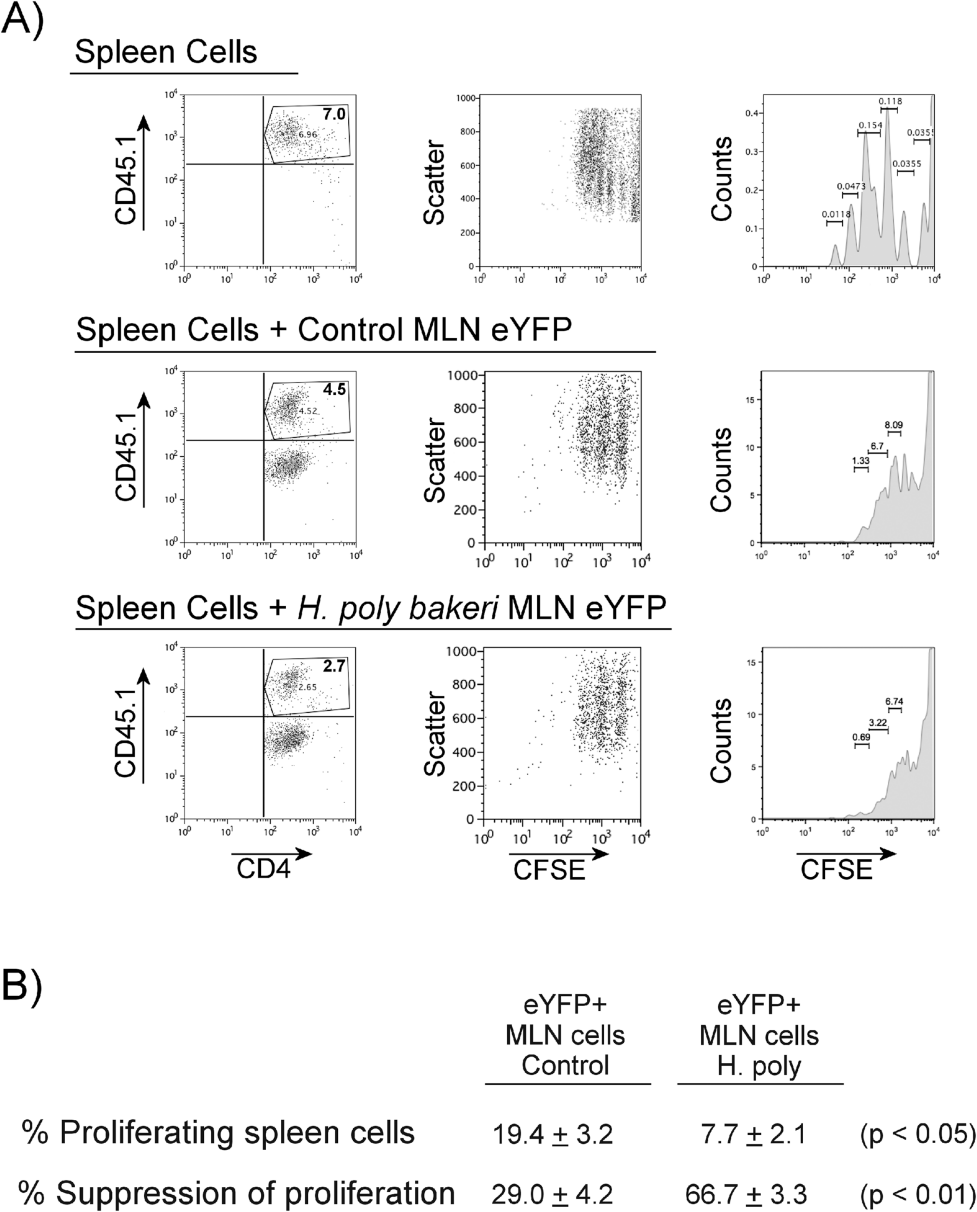
Helminth exposure alters Th17 lineage MLN cell ability to suppress lymphocyte proliferation *in vitro*. eYFP+ MLN cells from helminth-naïve (Control) or H. polygyrus bakeri–infected mice were isolated by flow cytometry and added (in 1:2 ratio) to cultures of CFSE-labeled splenic T cells stimulated with anti-CD3. **(A)** Representative results showing suppression of splenic T proliferation by addition of eYFP+ MLN. **(B)** Table shows means ± S.E. from three independent experiments.

Suppressor T cells that express Foxp3 can inhibit proliferation of mitogen-stimulated splenic T cells. Because helminth infection increased the percentage of MLN Th17 lineage cell Foxp3 expression, we examined if this was associated with increased suppressor T-cell function. As shown in **Figure 6A**, addition of eYFP-expressing T cells isolated from the MLN of helminth naïve mice suppressed mitogen-stimulated proliferation of CFSE-labeled splenic T cells. However, addition of equal numbers of eYFP-expressing T cells from MLN pf *Hpb*-infected mice, rather than eYFP-expressing cells from helminth-naïve mice, was much more effective (>2-fold, **Figure 6B**) in reducing splenic T-cell proliferation.

### Co-transfer of Th17 lineage cells from helminth-infected mice suppress T-cell transfer colitis

**Figure 7:**
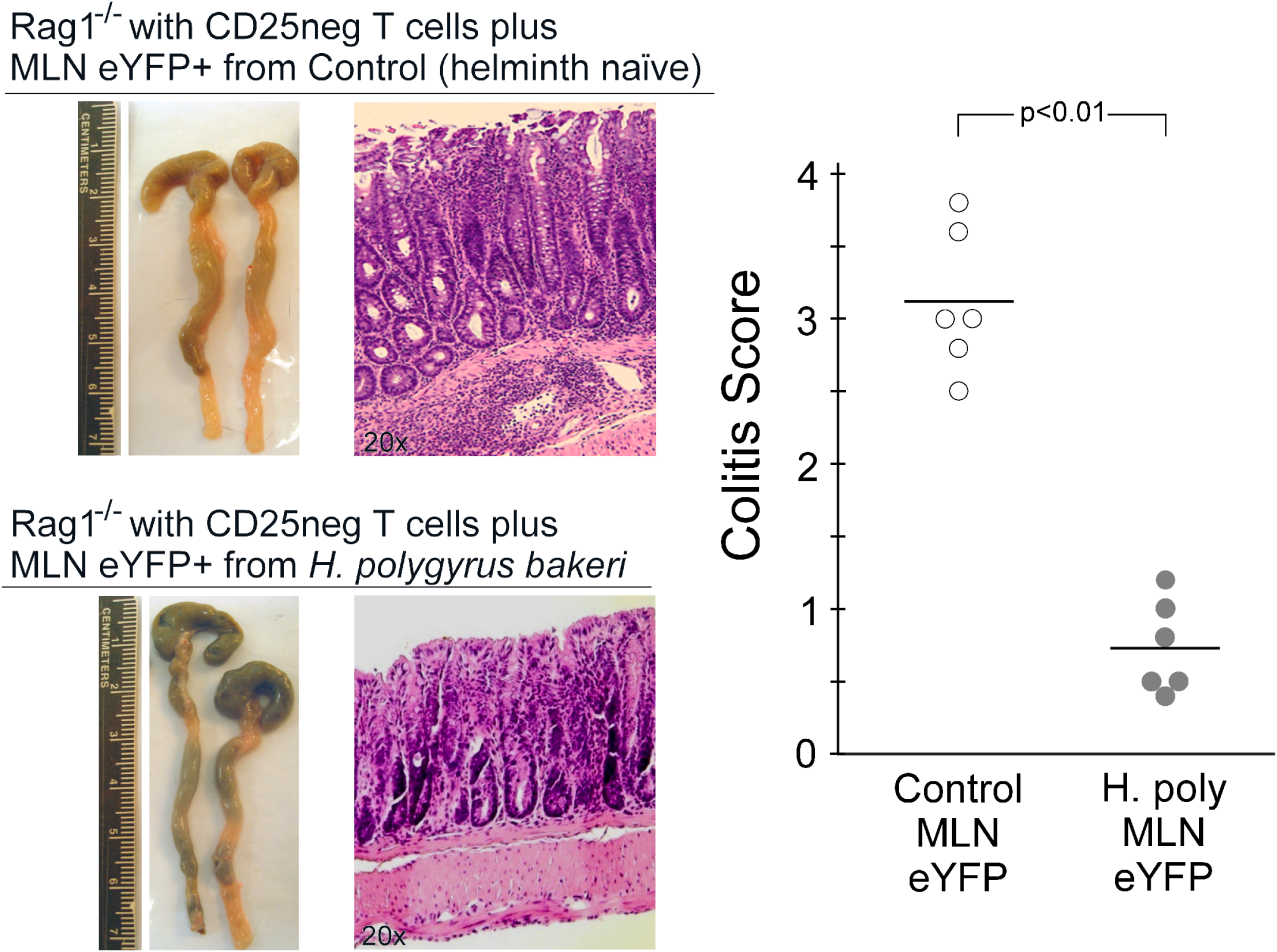
Co-transfer of Th17 lineage MLN cells from mice exposed to *H. polygyrus bakeri* suppress T cell transfer colitis. Rag-1^−/−^ mice were reconstituted with a mixture of CD25-negative T cells from CD45.1 mice (5 × 10^6^ cells) and eYFP+ MLN cells (3 × 10^4^ cells) isolated from helminth naïve or *H. polygyrus bakeri*-infected mice. Following reconstitution, mice were treated with NSAID (piroxicam 80 mg/250 g of ground feed) for two weeks and sacrificed two weeks later. Representative gross colon and histology shown. Scatter graph shows individual colitis scores from three independent experiments with two mice per group per experiment. Significance determined by Mann–Whitney U test.

**Figure 8:**
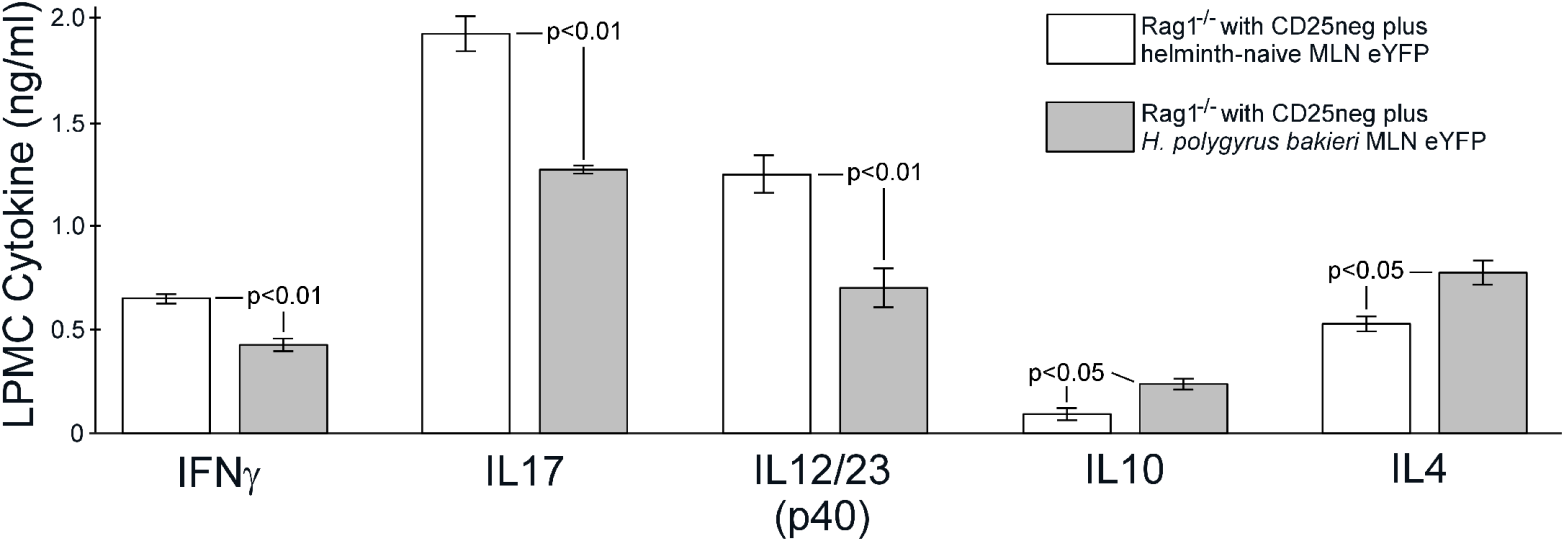
Co-transfer of Th17 lineage MLN cells from mice exposed to *H. polygyrus bakeri* alters LPMC cytokine expression in mice with T cell transfer colitis. LPMC were isolated from colons of Rag-1^−/−^ mice reconstituted with CD25neg splenic T cells co-transferred with flow-cytometry isolated MLN eYFP+ T cells from helminth naïve (open box) or *H. polygyrus bakeri*-infected (gray box) IL-17 reporter mice, stimulated with anti-CD3 (IFN-γ,IL-17a, IL-10, IL-4) or LPS (IL-12p40) and cultured for 48h. Culture supernatants were analyzed for cytokines by ELISA. Data are means ± SE from three independent experiments.

Helminth infection increases the percentage of IL-10 and Foxp3 expression by the remaining Th17 lineage cells and augments suppressor cell activity as measured by reduction in T cell proliferation. This suggests that helminth infection may make Th17 cells function like regulatory T cells. Immunodeficient mice develop severe colitis when reconstituted with CD25-negative T cells. This murine T-cell transfer model of colitis immunologically resembles human Crohn’s disease (18). Co-transfer of regulatory T cells along with the CD25neg T cells can suppress T-cell transfer colitis. Therefore, to test their regulatory function, we co-transferred eYFP-expressing T cells isolated from the MLN of either helminth-naïve or *Hpb*-infected mice along with CD25neg T cells into Rag-1-deficient mice and measured the development of colitis. Severe colitis developed in Rag-1-deficient mice reconstituted with CD25neg T cells and eYFP-expressing MLN cells from helminth-naïve mice (**Figure 7**). Significantly milder colitis developed in Rag-1 mice reconstituted with CD25neg T cells and eYFP-expressing MLN cells isolated from *Hpb*-infected mice. We also examined cytokine expression by cultured LPMCs isolated from the intestine of these mice with T-cell transfer colitis as measured by ELISA (**Figure 8**). Consistent with the reduction in severity of colitis, co-transfer of eYFP-expressing MLN cells from helminth-infected mice significantly reduced the mitogen-stimulated production of IFN-γ (helminth-naïve 0.65 ± 0.02 ng/ml, *Hpb*-infected 0.43 ± 0.03 ng/ml, mean ± SE, *p* < 0.01), IL-17a (helminth naïve 1.92 ± 0.08 ng/mL, *Hpb*-infected 1.27 ± 0.02 ng/ml, mean ± SE, *p* < 0.01), and IL-12p40 (helminth-naïve 1.25 ± 0.09 ng/ml, *Hpb*-infected 0.70 ± 0.09 ng/ml, mean ± SE, *p* < 0.01) from LPMC cells while significantly increasing their production of IL-10 (helminth-naïve 0.09 ± 0.02 ng/ml, *Hpb*-infected 0.24 ± 0.03 ng/ml, mean ± SE, *p* < 0.05), and IL-4 (helminth-naïve 0.53 ± 0.04 ng/ml, *Hpb*-infected 0.77 ± 0.06 ng/ml, mean ± SE, *p* < 0.05).

## Discussion

Helminth infections alter host immunity and can suppress host Th17 function (4, 11). Th17 cell activity drives many autoimmune and inflammatory diseases (19). However, Th17 cells are highly plastic; in some responses, they drive pathological inflammation, and other situations appear non-inflammatory or even regulatory (6). Because helminth infection can suppress pathogenic Th17-driven inflammation, we investigated whether exposure to a strictly intestinal helminth, *H. polygyrus bakeri* (*Hpb*), alters gut-associated Th17 cell plasticity. We inoculated C57BL/6 Th17 lineage reporter mice with *Hpb* 3rd stage larva. This is a nematode parasite that only colonizes the duodenum and proximal jejunum of rodents and does not migrate to or through other organs (20). Th17 lineage reporter mice are heterozygous for functional IL-17, as the gene in one locus is replaced with Cre recombinase (14). When bred to R26R^eYFP^ mice (15), activation of the native IL-17a promoter and gene expression in a cell also results in Cre-mediated excision of a floxed stop codon that then allows constitutive production of a yellow variant of green fluorescent protein in that cell. Thus, any cell that at one time significantly activated the IL-17a locus will persistently express eYFP. We evaluated eYFP-expressing mesenteric lymph node cells from helminth-naïve mice or mice that harbored adult Hpb parasites having been inoculated 2 weeks before lymph node harvest.

Infection with *Hpb* significantly reduces the percentage of MLN CD4+ lymphocytes that produce IL-17a. This decrease is reflected in a parallel decrease in cells that express eYFP. This means that IL-17a production isn’t just “turned off” in Th17 cells but rather that there is a decrease in the frequency of Th17 lineage cells. However, we did find that IL-17a production is significantly suppressed in the remaining Th17 lineage cells. The decrease in percentage of cells that ever engaged the IL-17a locus and IL-17a production by cells that had previously activated that locus results in the profound drop in MLN IL-17a release following Hpb infection.

Pathogenic Th17 cells co-express IFN-γ and can effectively help drive several autoimmune and/or inflammation-mediated diseases like IBD (5, 21). We found that enteric *Hpb* infection significantly decreased the frequency to Th-17 lineage cells that make IFN-γ (**Figure 3**). This shift from a pathogenic Th17 subtype may account for much of the protection against inflammation we see in mouse models of IBD (16, 22, 23). However, the Th17 lineage is further skewed toward a potentially regulatory phenotype. We found that following *in-vivo* helminth exposure, a much higher percentage of Th17 lineage MLN cells make IL-10 as compared to the Th17 lineage MLN cells from helminth-naïve mice. This finding expands on the previously demonstrated expansion of IL-10-producing Th17 lineage cells that occurs on repeated exposure to *Nippostrongylus brasiliensis* (24) in that an additional helminth (*Hpb*) causes similar expansion but without the multi-organ involvement or need for repeated infection. IL-10-producing Th17 cells contribute to the regulation of inflammation (25, 26). Therefore, infection with Hpb, and perhaps helminth infection in general, appears to skew Th17 cells away from an inflammatory/pathogenic phenotype cytokine profile and toward a regulatory phenotype cytokine profile.

To further evaluate skewing of Th17 lineage MLN cells to a potentially regulatory phenotype, we tested their expression of Foxp3. Expression of the transcription factor Foxp3 identifies regulatory T cells (27) and is required for their differentiation and function. Double-expressing IL-17a+Foxp3+ GALT lymphocytes are well-described (28–30), but their function is not well-defined. Clones of CD4+IL-17a+Foxp3+ T cells could be expanded from a gut-associated lymphocyte population isolated from patients with Crohn’s disease but not from healthy controls (patients with colon cancer) or patients with ulcerative colitis (30). Those clones also expressed IFN-γ, and the authors speculated that they may help drive inflammation in Crohn’s disease. Others found increased frequency of CD4+IL-17a+Foxp3+ double-expressing lymphocytes in the peripheral circulation of patients with active Crohn’s disease and ulcerative colitis as compared to healthy controls (28). Yet, the peripheral Treg compartment in the patients with Crohn’s disease or ulcerative colitis appeared to have impaired regulatory capacity. We found that helminth infection doubles the percentage of Th17 lineage MLN cells that express the Foxp3 transcription factor. Helminth infection dramatically increases the percentage of Foxp3-expressing CD4+ T cells (16, 17). We examined if this helminth-associated increase in MLN Foxp3 expression was due to skewing or recruitment of Th17 lineage MLN cells or if they developed independently of the Th17 compartment. We found that while the percentage of Foxp3-expressing Th17 lineage MLN cells increased with *Hpb* exposure, the percentage of Foxp3 cells that co-expressed eYFP was decreased by fourfold (**Figure 5**). The decrease in the frequency of Th17-lineage MLN cells that occurs with infection also holds true in the Foxp3+ compartment. The expansion of the Foxp3+ compartment is not due simply to the skewed plasticity of Th17 cells.

Although not directly contributing much to the dramatic expansion of the Foxp3+ Treg MLN cells, the helminth-associated increased expression of the Foxp3 transcription factor in Th17 lineage cells is congruent with increased IL-10 and decreased IFN-γ expression. We next tested if Th17 lineage MLN cells from helminth-infected mice had regulatory rather than inflammatory activity.

Classical Treg cells suppress *in vitro* proliferation of mitogen-stimulated T cells (13). We found that following *Hpb* infection, MLN Th17 lineage cells acquire this suppressive activity as compared to Th17 lineage cells from helminth-naïve mice. We also tested their *in-vivo* regulatory function using a T-cell transfer colitis model (16). We found that co-transfer of 3.0 × 10^4^ MLN eYFP+ cells from helminth-naïve mice along with 5.0 × 10^5^ colitigenic CD25neg T cells into Rag-1 mice permitted the development of severe intestinal inflammation. Therefore, even though the Th17 lineage cells from helminth-naïve mice remained heterogeneous for a functional IL-17 gene, they did not inhibit the development of colitis. However, co-transfer of 3.0 × 10^4^ MLN eYFP+ cells from helminth-infected mice along with 5 × 10^5^ colitogenic CD25neg T cells into Rag-1 mice significantly attenuated the development of colitis. Thus, helminth infection imparted a regulatory tone to MLN Th17 lineage cells. The transferred Th17 lineage cells from helminth-infected mice contained 2.25 times more Foxp3+ T cells than MLN eYFP+ cells form helminth-naïve mice. However, although expanded, this remains a small absolute number (~2400) of transferred Foxp3+ T cells. The transferred Th17 lineage cells contained 5.48 times more IL-10-expressing T cells than MLN eYFP+ cells from helminth naïve mice. It may be that this skewing, in addition to the increased percentage of Foxp3+ T cells, helps impart regulatory tone. Indeed, this skewing of the Th17 compartment had a dramatic effect on the co-transferred CD25neg cells and colonic innate cells. The colonic LPMC from mice given MLN Th17 lineage cells from *Hpb*-infected mice had a diminished inflammatory cytokine profile.

As locales adopt increasingly hygienic lifestyles, they avoid previously common pathogenic infections like those caused by helminths. Unfortunately, concurrent with this adoption there is also an increase in autoimmune and inflammatory disease (1). We are studying how loss of previously ubiquitous helminth infection may augment the risk for developing poorly regulated pathogenic inflammation like that caused by excessive Th17 responses. We find that in addition to suppressing the frequency of gut-associated Th17 cells, helminths also alter the plasticity of this cell compartment, skewing Th17 cells away from an inflammatory and toward a regulatory profile. This skewing could permit maintenance of homeostatic function (6) while preventing pathogenesis, effectively dulling one side of a two-edged sword (7).

## Data Availability

The raw data supporting the conclusions of this article will be made available by the authors, without undue reservation.
