# Supplementary figures and images for: Helminth exposure influences Th17 plasticity, suppressing inflammatory and promoting regulatory activity by Th17 lineage cells
Source: Front Immunol. 2026 Apr 28;17:1767639. doi: 10.3389/fimmu.2026.1767639 (PMC13161039; doi:10.3389/fimmu.2026.1767639)

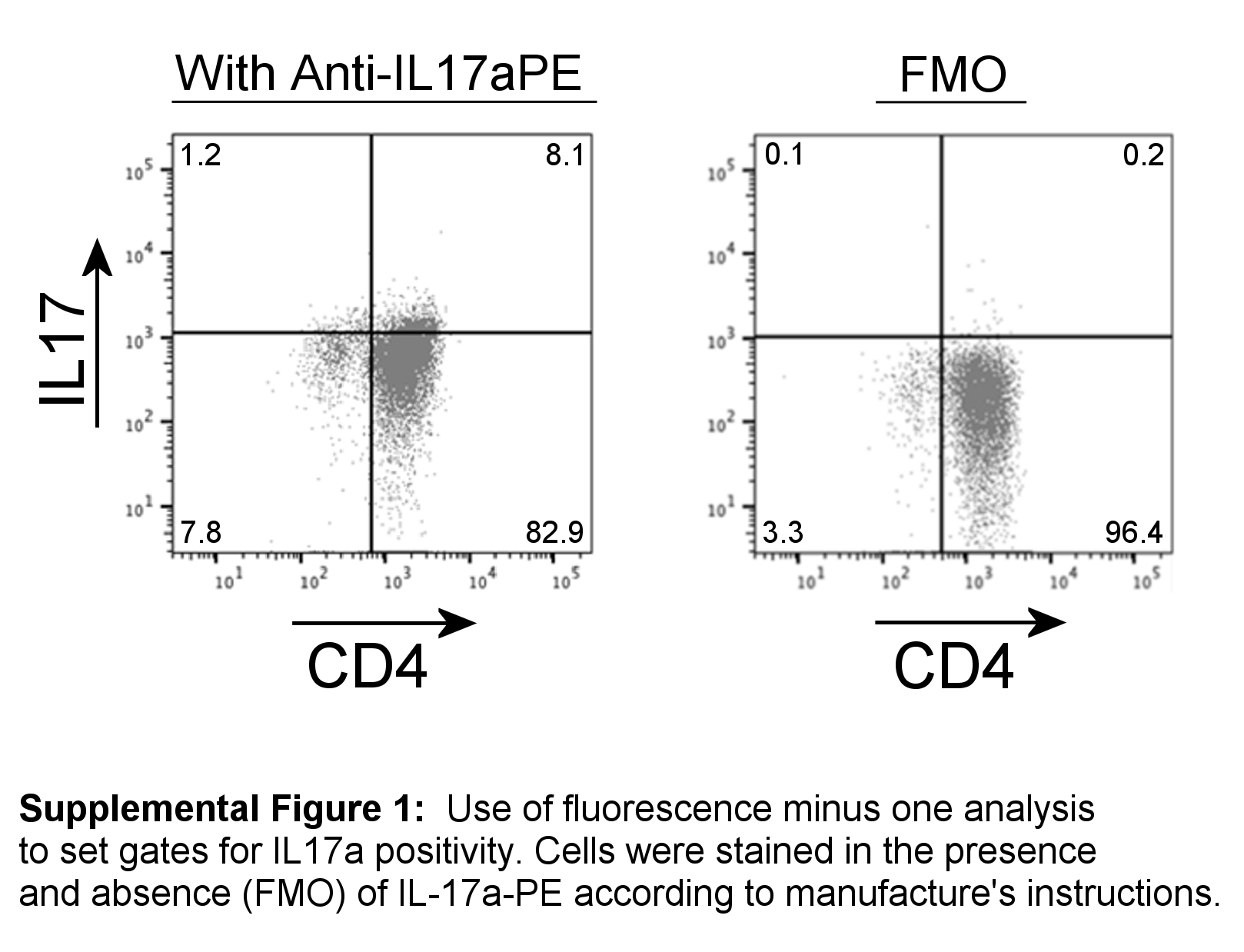

Supplement: Supplementary file 1 [file Image1.tif]
